# Dendritic cell-mediated chronic low-grade inflammation is regulated by the RAGE-TLR4-PKCβ_1_ signaling pathway in diabetic atherosclerosis

**DOI:** 10.1186/s10020-022-00431-6

**Published:** 2022-01-21

**Authors:** Liding Zhao, Ya Li, Tian Xu, Qingbo Lv, Xukun Bi, Xianglan Liu, Guosheng Fu, Yunzeng Zou, Junbo Ge, Zhaoyang Chen, Wenbin Zhang

**Affiliations:** 1 Department of Cardiovascular Diseases, Sir Run Run Shaw Hospital, College of Medicine, Zhejiang University, No 3 East of Qinchun Road, Hangzhou, Zhejiang 310000 People’s Republic of China; 2 Key Laboratory of Cardiovascular Intervention and Regenerative Medicine of Zhejiang Province, Hangzhou, Zhejiang People’s Republic of China; 3 Shanghai Institute of Cardiovascular Diseases of Zhongshan Hospital, Fudan University, Shanghai, China; 4 Institute of Biomedical Science, Fudan University, Shanghai, China; 5 Heart Center of Fujian Province, Union Hospital, Fujian Medical University, 29 Xin-Quan Road, Fuzhou, 350001 People’s Republic of China

**Keywords:** Atherosclerosis, Diabetes, Inflammation, Dendritic cells, Protein Kinase C

## Abstract

**Background:**

The unique mechanism of diabetic atherosclerosis has been a central research focus. Previous literature has reported that the inflammatory response mediated by dendritic cells (DCs) plays a vital role in the progression of atherosclerosis. The objective of the study was to explore the role of DCs in diabetes mellitus complicated by atherosclerosis.

**Methods:**

ApoE^−/−^ mice and bone marrow-derived DCs were used for in vivo and in vitro experiments, respectively. Masson’s staining and Oil-red-O staining were performed for atherosclerotic lesion assessment. The content of macrophages and DCs in plaque was visualized by immunohistochemistry. The expression of CD83 and CD86 were detected by flow cytometry. The fluctuations in the RNA levels of cytokines, chemokines, chemokine receptors and adhesions were analyzed by quantitative RT-PCR. The concentrations of IFN-γ and TNF-α were calculated using ELISA kits and the proteins were detected using western blot. Coimmunoprecipitation was used to detect protein–protein interactions**.**

**Results:**

Compared with the ApoE^−/−^ group, the volume of atherosclerotic plaques in the aortic root of diabetic ApoE^−/−^ mice was significantly increased, numbers of macrophages and DCs were increased, and the collagen content in plaques decreased. The expression of CD83 and CD86 were significantly upregulated in splenic CD11c^+^ DCs derived from mice with hyperglycemia. Increased secretion of cytokines, chemokines, chemokine receptors, intercellular cell adhesion molecule (ICAM), and vascular cell adhesion molecule (VCAM) also were observed. The stimulation of advanced glycation end products plus oxidized low-density lipoprotein, in cultured BMDCs, further activated toll-like receptor 4, protein kinase C and receptor of AGEs, and induced immune maturation of DCs through the RAGE-TLR4-PKCβ_1_ signaling pathway that was bound together by intrinsic structures on the cell membrane. Administering LY333531 significantly increased the body weight of diabetic ApoE^−/−^ mice, inhibited the immune maturation of spleen DCs, and reduced atherosclerotic plaques in diabetic ApoE^−/−^ mice. Furthermore, the number of DCs and macrophages in atherosclerotic plaques was significantly reduced in the LY333531 group, and the collagen content was increased.

**Conclusions:**

Diabetes mellitus aggravates chronic inflammation, and promotes atherosclerotic plaques in conjunction with hyperlipidemia, which at least in part through inducing the immune maturation of DCs, and its possible mechanism of action is through the RAGE-TLR4-pPKCβ_1_ signaling pathway.

**Supplementary Information:**

The online version contains supplementary material available at 10.1186/s10020-022-00431-6.

## Introduction

The risk of coronary heart disease in individuals with diabetes mellitus (DM) is two to six times greater than for individuals without DM (Sasso et al. 2004). Previous studies have reported that increased secretion of tumor necrosis factor α (TNFα), interleukin (IL)-6, C-reactive protein (CRP), and other substances at low concentrations lead to chronic low-grade inflammatory responses accompanied by the development and progression of diabetes mellitus (Castelblanco et al. 2018). This inflammatory mechanism is currently considered to be a leading mechanism for the development and progression of atherosclerosis (Zhang et al. 2019). The hyperglycemic state of diabetes accelerates atherosclerosis in conjunction with hyperlipidemia, but the mechanism is still not well understood (Wu and Huan 2007). When there is the appearance of atherosclerotic plaque together with diabetes mellitus, atherosclerosis presents several distinctive characteristics, such as microvascular lesions and diffuse lesion plaques, which illustrates that the development of atherosclerosis in DM patients has its own specific mechanism (Coutant et al. 2004).

It is believed that DCs, specialized antigen-presenting, and immune inflammatory-response-initiating cells, plays a role as a "bidirectional immune regulator" in the occurrence and development of atherosclerosis (Daissormont et al. 2011; Kanter et al. 2012). The degree of atherosclerosis that is produced may depend on the balance of immune regulation (Daissormont et al. 2011; Kanter et al. 2012). DCs secrete a range of cytokines, chemokine and chemokine receptors (CCL4, CCR7, CXCR4) after immune maturation to respond to the stimulation of antigens or immune inflammation (Ssemakalu et al. 2015). Cytokines that induce inflammation, such as IL1, IL-6, IL-12, and TNF-α are referred to as pro-inflammatory cytokines (Dinarello 2000). Cytokines such as IL-4 and IL-10 that inhibit the inflammatory processes are called anti-inflammatory cytokines. Previous studies have shown that a high-glucose (Cao et al. 2015) and high-insulin (Lu et al. 2015) environment can promote the differentiation and immune maturation of DCs. Injecting diphtheria toxin into DTR^+/+^LDLR^−/−^mice to induce apoptosis of DCs can reduce plaque formation by 55%, and enhance plaque stability (Paulson et al. 2010). These results indicate that reducing DC-mediated inflammation is beneficial in reducing atherosclerosis. Since there is currently no direct way to reduce the number of DCs in clinical practice, regulation of DC maturation, and reduction of the release of inflammatory factors is worthy of attention. Previous studies found that the PKC signaling pathway is not only involved in the formation of atherosclerotic plaques (Durpes et al. 2015), but also plays an essential role in the differentiation and immune maturation of DCs (Stein et al. 2017). However, there is limited data to suggest the role PKCs in atherosclerosis under hyperglycemic condition, and, therefore, the DC maturation elicited in diabetic atherosclerosis.

In this study, we demonstrated that activation of the RAGE-TLR4-PKCβ_1_ signaling pathway in a high-glucose and high-fat environment was involved in the immune maturation of DCs and the occurrence and development of chronic low-grade inflammation in diabetes, which was closely related to diabetic atherosclerosis.

## Material and methods

### Animals and splenic CD11c^+^ DCs separation

All experimental animal procedures were approved by the Animal Care and Use Committee of Fudan University. ApoE^−/−^ mice (Beijing Vital River Laboratory Animal Technology Co., Ltd. male, aged eight weeks and weighing approximately 20 g) were housed in the Animal Administration Center of Fudan University, Shanghai, China. Mice were maintained in cages with a maximum of four mice per cage. The mice were randomly divided into a diabetic group and a control group. Mice in the diabetic group received an intraperitoneal injection of streptozotocin (STZ, 80 mg/kg, Sigma, St Louis, MO, USA) daily for one week. Then the mice were maintained under the same conditions for an additional week before their blood glucose levels were monitored by collecting blood from the tail vein. Mice with fasting blood glucose levels over 13 mM were deemed diabetic and fed a normal diet for eight weeks. Additionally, ApoE^−/−^ mice, aged eight weeks and weighing approximately 20 g, were treated with STZ in the same way as described above, then randomly divided into a diabetic group and an LY333531 group. Each mouse was gavaged once a day with 200μL 10% dimethyl sulfoxide for eight weeks. Half of the mice received 1 mg/kg LY333531 with 10% dimethyl sulfoxide, and half did not. The body weights were measured at the end of eight weeks. At the end of the experiment, the animals were euthanized using CO_2_ inhalation, the mice eyeballs were extracted and whole blood was taken. The blood lipid profile, such as total cholesterol (TC), low-density lipoprotein cholesterol (LDL-C), high-density lipoprotein cholesterol (HDL-C), and triglycerides (TG), were measured by the Nanjing Jiancheng Bioengineering Institute. The spleens were removed, crushed, and the CD11c^+^ DCs were isolated using anti-CD11c^+^ microbeads (Miltenyi Biotec, Bergisch Gladbach, Germany). The experiment designer was aware of the group allocation while data collectors and data analysts were not during the outcome assessment and data analysis.

### Organ collection and processing

The aorta and heart were carefully perfused with physiological saline to remove the blood. The heart apex was removed, and the aortic arch was separated from aorta distal to the left subclavian artery. The isolated tissues were embedded in optimal cutting temperature compound (OCT compound) and frozen, then stored at -80℃.

### Atherosclerotic lesion assessment

The frozen aortic roots were sectioned at a thickness of eight µm using a CM3050S cryostat (Leica Biosystems). Oil-red-O staining was to visualize plaque extension in the aortic root. Frozen sections were fixed, washed, and incubated for 18 min in an Oil-red-O solution, then counterstained with 0.25% brilliant green (Sinopharm Chemical Reagent, China) for 6 min. Oil-red-O staining was used to determine the lipid content of the plaques. Masson’s staining was performed using a Masson kit (Sinopharm Chemical Reagent, China) to stain collagen present in the atherosclerotic lesions. Images were obtained with an optical microscope (Olympus, Japan). Images were analyzed, and quantification was performed using NIH Image J software (Schneider et al. 2012).

### Immunohistochemistry

To visualize macrophages in the plaque area, paraformaldehyde-fixed aortic root sections were stained with CD68-FITC antibody (1:50, MA5-16676, Invitrogen) using 4',6-diamidino-2-phenylindole (DAPI) (2ug/ml, D9542, Sigma, USA) as a nuclear counterstain. The presence of DCs in the plaques also was visualized. Paraformaldehyde-fixed sections of aortic root were stained with CD11c antibody (1:100, ab33483, Abcam, USA) and a hamster secondary antibody (PE, 1:100, 12-4112-83, Invitrogen) using DAPI (2ug/ml, D9542, Sigma, USA) as a nuclear counterstain. The co-staining of CD68 and CD14 with CD11c was conducted with CD11c antibody (1:100, Huabio, cat.no. RT1108, lot.no. HL1212, China) and a mouse secondary antibody (1:500, Invitrogen, cat.no. A32723), CD68 antibody (1:100, Servicebio, cat.no. GB11067, lot.no. AC2103065A), CD14 antibody (1:100, Abcam, cat.no. ab221678, lot.no. GR3264380-5), and a rabbit secondary antibody (1:500, Invitrogen, cat.no. A32740). The co-staining of PKC beta isoforms and RAGE with CD11c was conducted with CD11c antibody (1:100, Huabio, cat.no. RT1108, lot.no. HL1212, China) and a mouse secondary antibody (1:500, Invitrogen, cat.no. A32727), PKCβ antibody (1:100, Abclonal, cat.no. A13628, lot.no. 5500002416, China), RAGE antibody (1:50, Abcam, cat.no. ab216329, lot.no. GR3274230-10, USA), and a rabbit secondary antibody (1:500, Invitrogen, cat.no. A32731). Positive-stained areas were selected by an investigator blinded to the treatment, observed using a fluorescence microscope (Olympus, Japan), and quantified using NIH Image J software (Schneider et al. 2012).

### Cell culture

Bone marrow-derived DCs (BMDCs) were obtained from C57 BL/6 mice (Beijing Vital River Laboratory Animal Technology Co., Ltd.). After the mice were euthanized using CO_2_ inhalation, the femurs of both pelvic limbs were removed, and the bone marrow cavity was flushed to obtain bone marrow cells. Bone marrow progenitor cells were cultured in Roswell Park Memorial Institute (RPMI) 1640 medium containing 10 ng/ml granulocyte–macrophage colony-stimulating factor (GM-CSF) (R & D Systems, Minneapolis, MN, USA) and 1 ng/ml IL-4 (R & D Systems, Minneapolis, MN, USA). The non-adherent cells were gently washed out after 24 h, and the remaining loosely adherent clusters were cultured. The culture medium was changed every other day. On day six, the cells were stimulated using 50ug/ml oxidized low-density lipoprotein (oxLDL) (Serotec, UK) for 48 h, with or without 200 ug/ml AGEs-BSA (BioVision, Palo Alto, CA, USA) interference to simulate the high-lipid and high-glucose environment of ApoE^−/−^ mice, respectively.

### Flow cytometry

Splenic DCs and BMDCs (1 × 10^6^) were harvested and washed, followed by incubation with CD83-PE-conjugated and CD86-FITC-conjugated (BD Pharmingen, San Diego, CA, USA) monoclonal antibodies for 30 min at 4℃. Then the cells were washed and analyzed using a flow cytometer (BD Bioscience). Cells stained with the appropriate isotype-matched immunoglobulin were used as negative controls.

### Quantitative RT-PCR

Total RNA was extracted from splenic CD11c + DCs or BMDCs using Trizol reagent (Invitrogen, Carlsbad, CA, USA). We used a SYBR RT-PCR kit (Takara, Dalian, China) for quantitative RT-PCR analyses. The primers for the cytokine genes (IL10, TNFα, IL12a, IL12b, IL1b, IL4, and IL6), chemokine and chemokine receptor genes (CCL4, CCR7, and CXCR4), adhesion genes (ICAM and VCAM), and GAPDH are listed in Additional file 1: Table S1. The relative expression levels of the genes were normalized to those of GAPDH using the 2^−ΔΔCt^ cycle threshold.

### Quantification of cytokine production by ELISA

The cells and supernatant of BMDCs were harvested and stored at -80℃ until they were assayed. The concentration of CRP and cytokine (interferon-γ (IFNγ) and TNFα) were analyzed using ELISA kits according to the manufacturer’s instructions (R&D Systems, Minneapolis, MN, USA).

### Coimmunoprecipitation

Extracts from mouse BMDCs were prepared in lysis buffer (50 mmol/L Tris, pH 7.5, 150 mmol/L NaCl, 1% v/v Sigma Protease and Phosphatase Inhibitor Cocktail) and diluted to a final volume of 500μL (500 μg). Protein A:protein G beads (1:50; Sigma) were added, then polyclonal TLR4 antibody (2 μg; cat.no. 19811–1-AP, ProteinTech company) and polyclonal phospho-PKCβ_1_ antibody (2 μg, cat.no. PA5-105,463, Invitrogen) also were added, respectively. After 4-h of continuous gentle agitation at 4℃, the beads were collected by pulse spin, washed three times in lysis buffer, and resuspended in PBS.

### Western blot

BMDCs were harvested for the extraction of total proteins with RIPA buffer. The protein concentrations were determined using a BCA protein assay (Beyotime, Shanghai, China). Antibodies of toll-like receptor 4 (TLR4) (1:1000, cat.no. 14358), RAGE (1:1000, cat.no. 55222), interleukin receptor-associated kinase 4 (IRAK4) (1:1000, cat.no. 4363), phosphor-IRAK4 (1:1000, cat.no. 11927), phospho-NF-κB (1:1000, cat.no. 3033), NF-κB (1:1000, cat.no. 8242), phospho-IκB (1:1000, cat.no. 9246), IκB (1:1000, cat.no. 9242) were all purchased from Cell Signal Technology, USA. Antibodies purchased from Santa Cruz included phospho-PKCα (1:300, cat.no. SC-377565), phospho-PKCβ_2_ (1:300, cat.no. SC-365463). Antibody of phospho-PKCβ_1_ was purchased from Invitrogen (1:500, cat.no. PA5-105463). Antibodies purchased from Abcam included phospho-PKCγ (1:1000, cat.no. ab5796), PKCα (1:1000, cat.no. ab32376), PKCβ_1_ (1:1000, cat.no. ab195039), PKCβ_2_ (1:1000, cat.no. ab32026), PKCγ (1:1000, cat.no. ab71558). The optical densities were analyzed using ImagePro 5.0 (Media Cybernetics, Inc., Silver Spring, MD, USA) and normalized to the protein level of β-actin or GAPDH.

### Statistical analysis

The results were presented as means ± S.E.M. and analyzed using one-way ANOVA followed by Tukey’s post-hoc test for multiple comparisons using the SPSS software package, version 16.0. P < 0.05 was considered to be statistically significant.

## Results

### Diabetic ApoE^−/−^ mice increased aortic atherosclerosis with chronic low-grade inflammation and dendritic cell activation

**Fig. 1:**
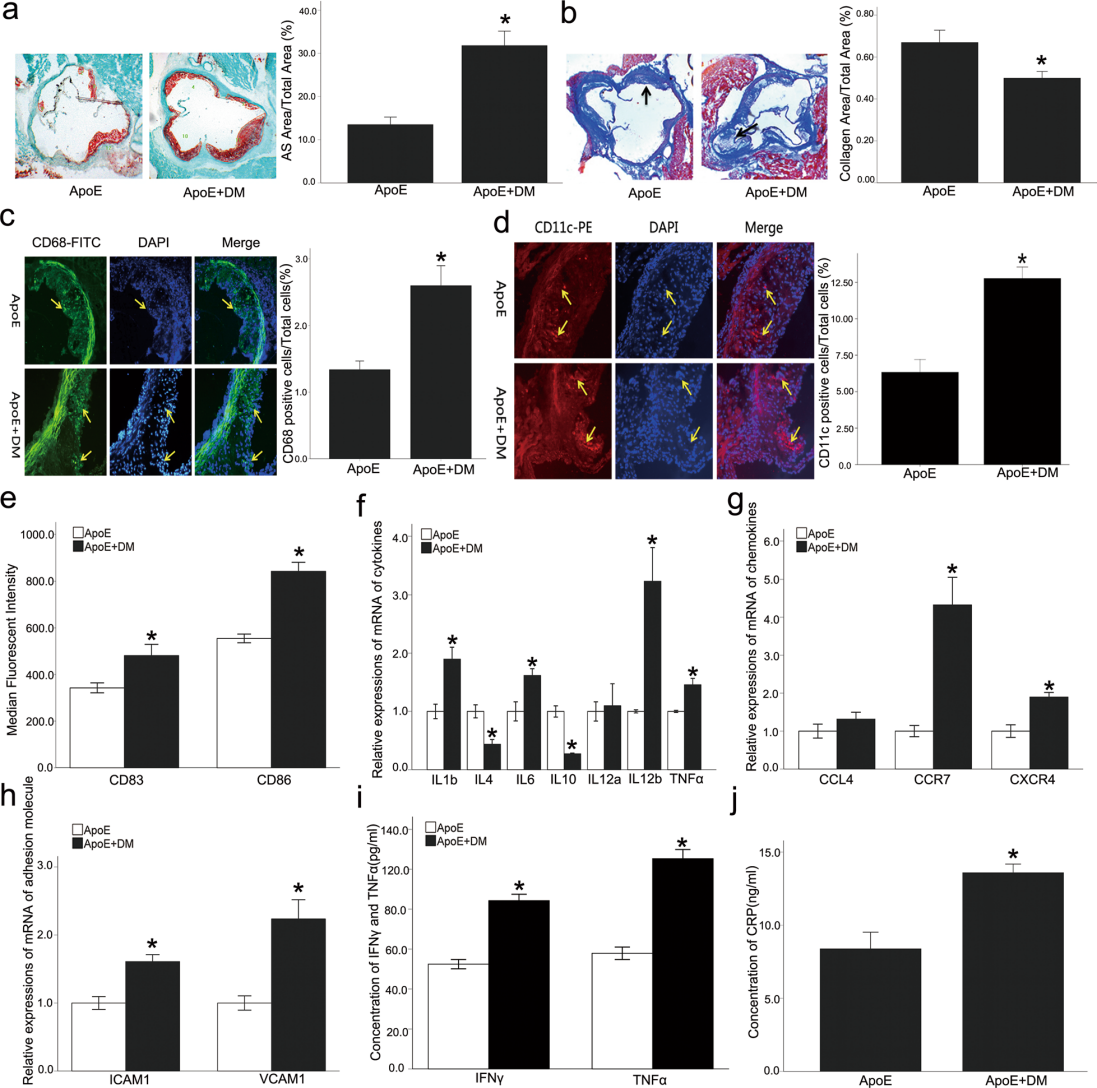
Diabetic ApoE^−/−^ mice increased aortic atherosclerosis with chronic low-grade inflammation and dendritic cell activation. Compared with the control group, the size of the atherosclerotic plaques (**a**) in the aortic roots of the diabetic ApoE^−/−^ mice was increased, and the plaque collagen content (**b**) was decreased, while the numbers of macrophages **c** and DCs **d** were increased. The expression of CD83 and CD86 **e** in DCs isolated from spleens was increased, as well as the expression of IL12b, IL4, IL6 (**f**), and chemokine receptors such as CCR7 and CXCR4 **g** also were up-regulated in the diabetic mice. The expression of ICAM and VCAM (**h**) in the aorta were up-regulated in the diabetic ApoE^−/−^ mice, and the concentrations of TNFα, IFNγ, and CRP (**i**, **j**) were also significantly increased in peripheral blood. Values, mean ± SED; n = 8; *p < 0.05 *vs*. DM group, scale bar, 250 μm; *DM* Diabetes mellitus, *AS* Atherosclerosis, *DAPI* 4,6-diamino-2-phenyl indole, *IL* Interleukin, *ICAM* Intercellular adhesion molecule, *VCAM* Vascular cell adhesion molecule, *TNFα* Tumor necrosis factor alpha, *IFNγ* IFN gamma. *CRP* C-reactive protein

The fasting blood glucose levels in the diabetic ApoE^−/−^ group were significantly increased, while the blood lipid levels were not significantly different (Additional file 1: Fig. S1). The thickness of the atheromatous plaque fibrous cap is reported to be proportional to the collagen content in it (Naghavi et al. 2003a, 2003b), and thicker fiber caps suggest more stable plaques. On the other hand, excessive aggregation of inflammatory cells such as macrophages and DCs indicates that plaques are more likely to rupture. The size of the atherosclerotic plaques in the aortic roots of the diabetic ApoE^−/−^ mice was significantly increased compared with the control group. The plaque collagen content was decreased, while the numbers of macrophages and DCs were increased (Fig. 1a–d). These results indicated that diabetes aggravated atherosclerosis independently of blood lipid levels and promoted inflammation within the plaque, which might decrease plaque stability. The expression of the co-stimulatory molecules, CD83 and CD86, in splenic DCs was significantly increased, and the expression of IL12b, IL4, IL6, and chemokine receptors such as CCR7 and CXCR4 also were significantly up-regulated in the diabetic mice (Fig. 1e–g). These results reveal that the atherosclerotic microenvironment associated with diabetes significantly promoted the immune maturation of DCs. The concentrations of TNFα, IFNγ, and CRP were significantly increased in peripheral blood, and the expression of ICAM and VCAM in the aorta also were up-regulated in the diabetic mice (Fig. 1h–j). These observations point to the existence of a chronic low-grade inflammatory response in the diabetic mice, which influenced the aortic adhesion molecule expression.

### AGEs plus oxLDL further induced the immune maturation of dendritic cells and activated the typical PKC signaling pathway

**Fig. 2:**
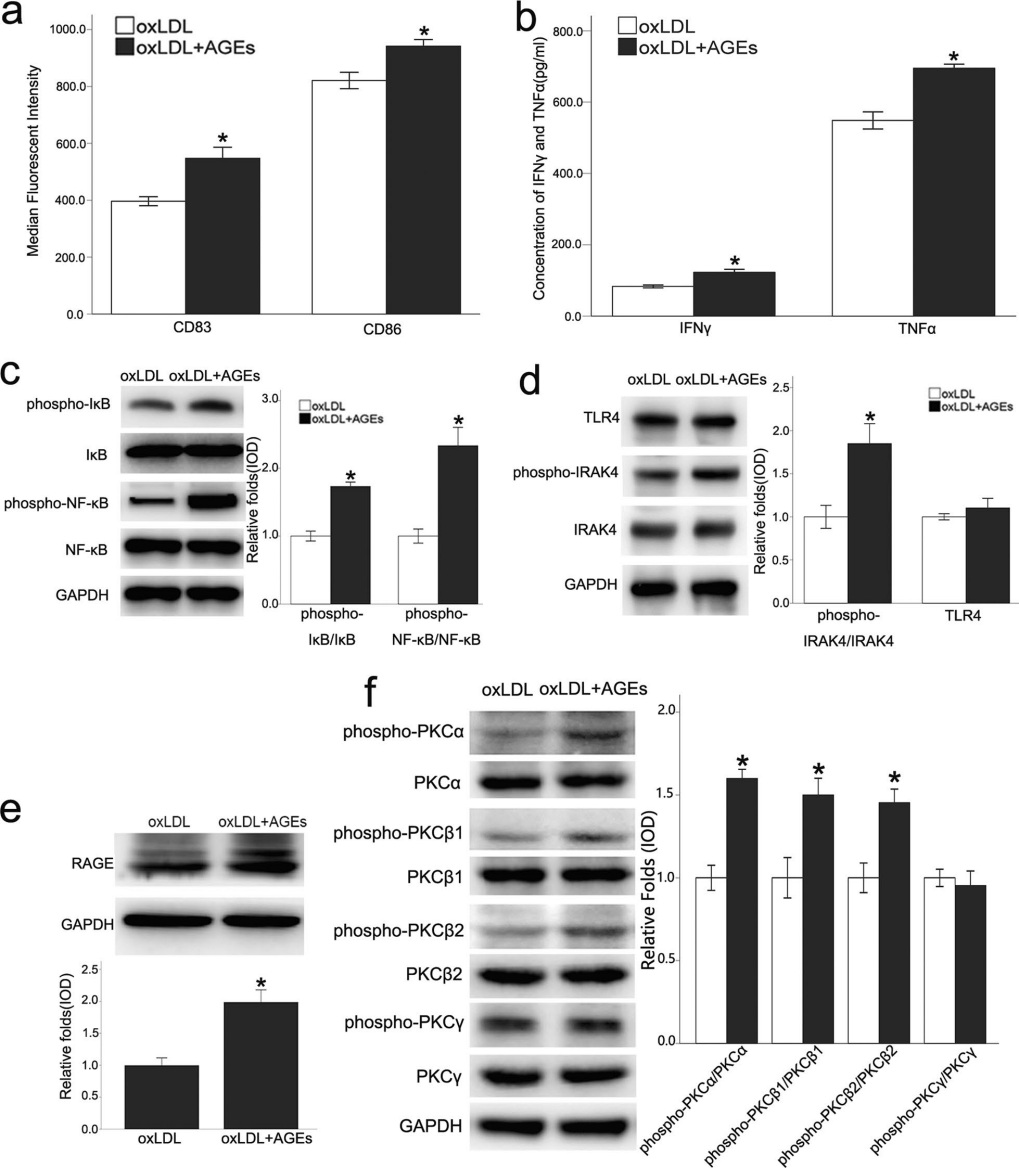
AGEs plus oxLDL further induced the maturation of DCs and activated certain signaling pathways. The oxLDL plus AGEs treatment increased the expression of CD83 and CD86 (**a**), in BMDCs, and also up-regulated expression of IFNγ and TNFα (**b**), which was demonstrated by ELISA. In conjunction with stimulation by oxLDL, AGEs induced a greater degree of the phosphorylation of IκB, NF-κB (**c**), IRAK4 (**d**), PKCα/β_1_/β_2_ (**f**), and the expression of RAGE (**e**). Values, mean ± SED; n = 3, *p < 0.05 *vs.* oxLDL group; *oxLDL* oxidized low density lipoprotein, *AGEs* advanced glycation end-products, *TNFα* Tumor necrosis factor alpha, *IFNγ* IFN gamma, *NF-κB* nuclear factor-κB, *RAGE* Receptor for advanced glycation end products, *pPKC* phosphorylated protein kinase C, *TLR4* Toll-like receptor 4, *IRAK4* Interleukin receptor associated kinase 4

After induction of differentiation, the number of CD11c^+^ cells detected by flow cytometry accounted for 78.4% of the total cells (Additional file 1: Fig. S2). Before using oxLDL, this study ruled out the presence of endotoxin contamination by limulus amebocyte lysate assay. In conjunction with stimulation by oxLDL, AGEs significantly induced expression of the maturation marker, CD83, and the co-stimulating molecule, CD86, in BMDCs. ELISA results also demonstrated significantly up-regulated expression of IFNγ and TNFα (Fig. 2a, b), suggesting that AGEs plus oxLDL promoted the release of inflammatory cytokines in BMDCs.

In addition, we found that oxLDL plus AGEs induced the phosphorylation of IκB and NF-κB (Fig. 2c) to a greater degree than oxLDL alone, and stimulated the nuclear transcription process. TLR4 is an important signal pathway for activating NF-κB, and it also is closely associated with DCs. AGEs plus oxLDL did not up-regulate the expression of TLR4. However, the phosphorylation of IRAK4, an important kinase downstream of TLR4 that is recruited by TLR4/MyD88, was significantly up-regulated (Fig. 2d), which would further phosphorylate IRAK1 and activate NF-κB. Previous studies found that various members of the PKC family are known to activate NF-κB (Oeckinghaus et al. 2011). This study focused on the typical PKCs instead of novel or atypical PKCs. In addition, as shown in this study, AGEs plus oxLDL can up-regulate the expression of RAGE, and activate PKCα and PKCβ_1_/β_2_ signaling pathways (Fig. 2e, f). Furthermore, the co-staining of PKC beta and RAGE with CD11c confirmed their co-localization in the aortic plaque (Additional file 1: Fig. S3a, b).

### PKC, RAGE, and TLR4 were involved in the immune maturation process of DCs with AGEs plus oxLDL intervention

**Fig. 3:**
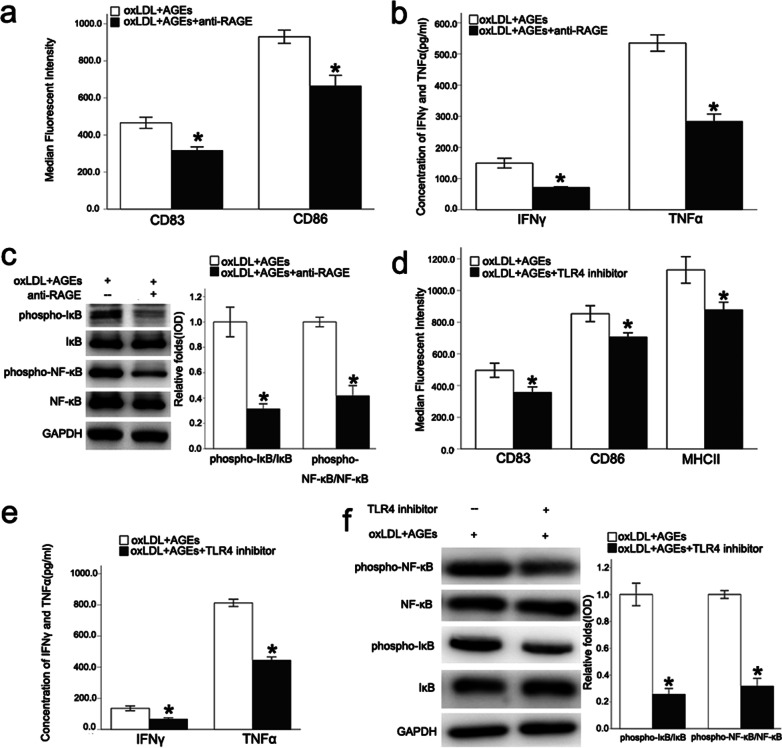
RAGE antibodies and TLR4 inhibitors inhibit the immune maturation of DCs. After pretreatment with RAGE neutralizing antibody, the expression of CD83 and CD86 were decreased, and accompanied by a significant down-regulation of secretion of the inflammatory cytokines and IκB/NF-κB phosphorylation (**a**–**c**). In the TLR4 inhibitor group, CD83 and CD86 were down-regulated, and the ability of BMDCs to release inflammatory cytokines also was decreased (**d**, **e**). Combined with the decreased expression of p-NF-κB and p-IκB (**f**). Values, mean ± SED; n = 3, *p < 0.05 *vs.* oxLDL + AGEs group; *oxLDL* oxidized low density lipoprotein, *AGEs* advanced glycation end-products; *RAGE* Receptor for advanced glycation end products, *NF-κB* nuclear factor-κB, *IFNγ* IFN gamma; *TLR4* Toll-like receptor 4, *TNFα* Tumor necrosis factor alpha

In this study, RAGE neutralizing antibody, TLR4 inhibitors, and three PKC inhibitors (CGP53353, Sigma-Aldrich, USA, 410 nM for PKCβII vs 3.8 μM for PKCβI vs 25uM for pKCα) that targeted corresponding PKC subtypes, were added to BMDCs with oxLDL plus AGEs. After pretreatment with RAGE neutralizing antibody, the expression of CD83 and CD86 were significantly decreased, and accompanied by a significant down-regulation of secretion of the inflammatory cytokines and IκB/NF-κB phosphorylation (Fig. 3a–c). These results suggested that the combination of AGEs and RAGE activated the immune maturation of BMDCs through the NF-κB signaling pathway.

In the TLR4 inhibitor group, CD83 and CD86 were significantly down-regulated, and the ability of BMDCs to release inflammatory cytokines also was decreased. Combined with the decreased expression of p-NF-κB and p-IκB (Fig. 3d–f), these observations indicated that the TLR4 signaling pathway was involved with oxLDL plus AGEs to induce the BMDCs immune maturation process.

**Fig. 4:**
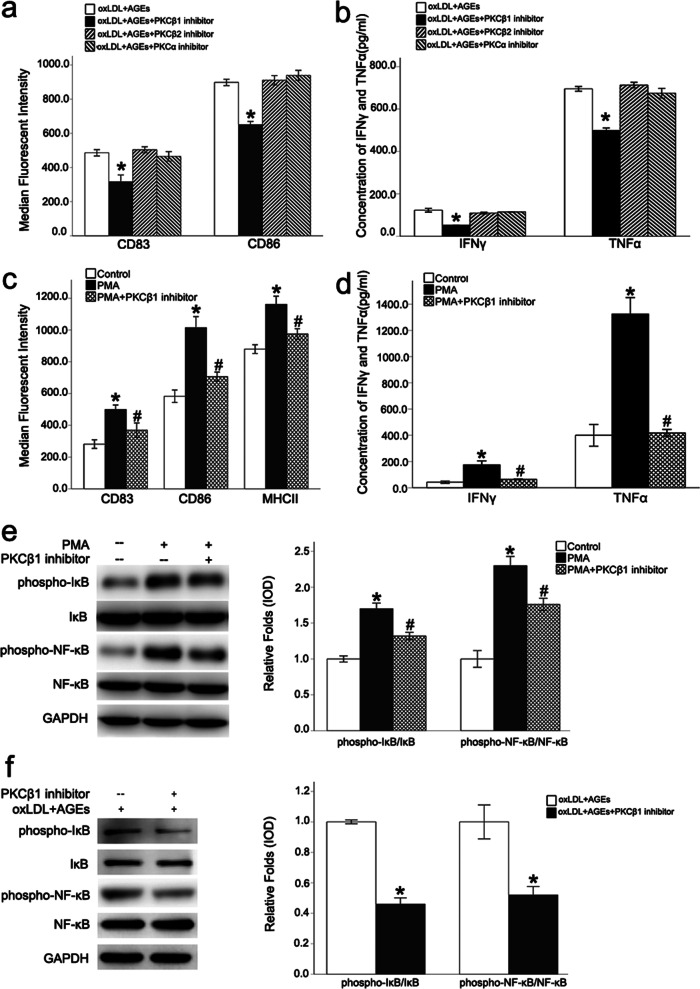
PKCβ_1_ inhibitors significantly inhibited the immune maturation of DCs. PKCβ_1_ inhibitors significantly down-regulated the expression of CD83 and CD86 in BMDCs, as well as the inflammatory cytokines, TNFα and IFNγ, tested by ELISA. However, PKCα inhibitors and PKCβ_2_ inhibitors did not have similar effects (**a**, **b**, *p < 0.05 *vs.* oxLDL + AGEs group). PMA, a PKC agonist, up-regulated the expression of CD83 and CD86 in BMDCs and promoted the secretion of the inflammatory cytokines, TNFα and IFNγ, while PKCβ_1_ inhibitors inhibited these effects (**c**, **d**, *p < 0.05 *vs.* control group; ^#^p < 0.05 *vs.* PMA group). PKCβ_1_ inhibitors inhibited the PMA− and oxLDL plus AGEs-induced activation of the NF-κB signaling pathway (**e**, **f**, *p < 0.05 *vs.* oxLDL + AGEs group). Values, mean ± SED; n = 3, *oxLDL* oxidized low density lipoprotein, *AGEs* advanced glycation end-products, *PKC* protein kinase C, *IFNγ* IFN gamma, *TNFα* Tumor necrosis factor alpha, *PMA* phorbol ester, *NF-κB* nuclear factor-κB

PKCβ_1_ inhibitors significantly down-regulated the expression of CD83 and CD86, as well as the inflammatory cytokines, TNFα and IFNγ, which are secreted by BMDCs. However, PKCα inhibitors and PKCβ_2_ inhibitors did not have similar effects (Fig. 4a, b). PMA, a PKC agonist, up-regulated the expression of CD83 and CD86 in BMDCs and promoted the secretion of the inflammatory cytokines, TNFα and IFNγ, while PKCβ_1_ inhibitors inhibited these effects (Fig. 4c, d). Additionally, PKCβ_1_ inhibitors inhibited the PMA- and oxLDL plus AGEs-induced activation of the NF-κB signaling pathway (Fig. 4e, f). This study repeated related experiments with PKCβ_1_ siRNA and found that the knockdown of PKCβ_1_ also inhibited the activation of NF-κB signaling pathway and the immune maturation of BMDCs (Additional file 1: Fig. S4), which is similar to the effect of PKC beta isoform 1 inhibitor. These results demonstrated that the PKCβ_1_ activity might have an essential role in the immune maturation of BMDCs.

### The relationship among RAGE, TLR4, and phospho-PKCβ_1_

**Fig. 5:**
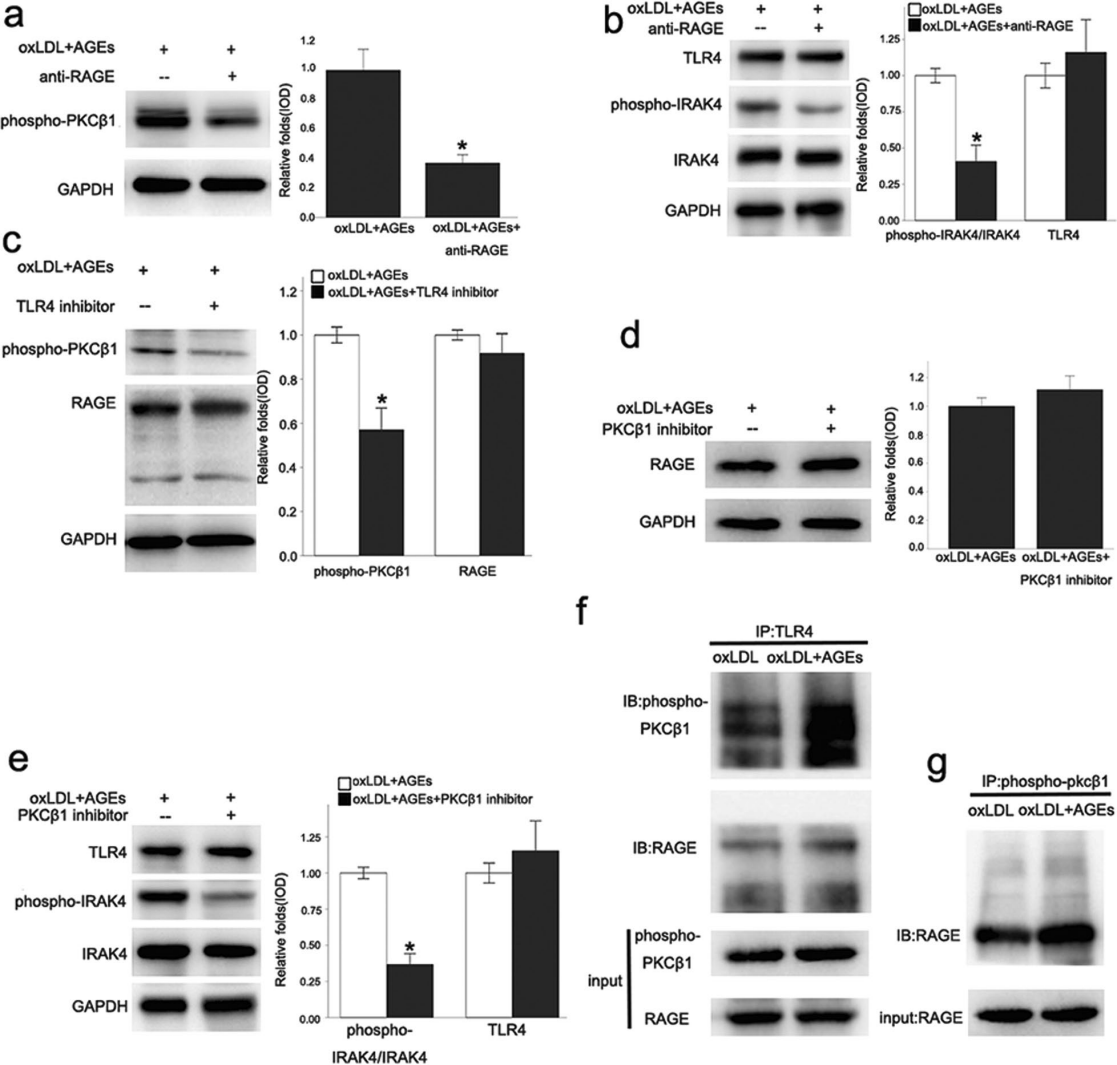
The relationship among RAGE, TLR4, and phospho-PKCβ_1_. The phosphorylation of PKCβ_1_, induced by oxLDL plus AGEs, was down-regulated by the RAGE neutralization antibody, as well as the phosphorylation of IRAK4, but there was no significant difference in the change in TLR4 (**a**, **b**). The phosphorylation of PKCβ_1_ was decreased in the TLR4 inhibitor group, while RAGE expression was not affected (**c**). TLR4 and RAGE expression in the PKCβ_1_ inhibitor intervention group showed no significant differences, but the phosphorylation of IRAK4 was significantly inhibited (**d**, **e**). Coimmunoprecipitation showed that TLR4 was bound to RAGE and phospho-PKCβ_1_ (**f**), while phospho-PKCβ_1_ was bound to RAGE (**g**). Values, mean ± SED; n = 3, *p < 0.05 *vs.* oxLDL + AGEs group, *oxLDL* oxidized low density lipoprotein, *AGEs* advanced glycation end-products, *RAGE* Receptor for advanced glycation end products, *PKC* protein kinase C, *TLR4* Toll-like receptor 4, *IRAK4* Interleukin receptor associated kinase 4

The phosphorylation of PKCβ_1_, which reflects the activity of PKCβ_1_, is induced by oxLDL plus AGEs, and it was significantly down-regulated by the RAGE neutralization antibody (Fig. 5a). However, after the inhibition of PKCβ_1_, there was no significant difference in the change in RAGE (Fig. 5d). This observation suggested that RAGE activation played an important role in the phosphorylation of PKCβ_1_, and PKCβ_1_ acted downstream of RAGE. The phosphorylation of PKCβ_1_ was significantly decreased in the TLR4 inhibitor group (Fig. 5c), while RAGE expression was not affected (Fig. 5c), which suggested that TLR4 is intermediate in the RAGE and PKCβ1 signal pathway. TLR4 expression in the PKCβ_1_ inhibitor intervention group showed no significant differences (Fig. 5e), suggesting that PKCβ_1_ might act downstream of TLR4. The involvement of TLR4 was required for the activation of PKCβ_1_ by RAGE. After the application of the PKCβ_1_ inhibitor or RAGE neutralizing antibody, phosphorylation of IRAK4 was significantly inhibited (Fig. 5b, e), which means that the TLR4/NF-κB signaling pathway was blocked and that PKCβ_1_ phosphorylation played a key role in the phosphorylation of IRAK4 induced by oxLDL plus AGEs. These results indicated that AGEs combined with RAGE and then activated the TLR4-PKCβ1 signaling pathway. Coimmunoprecipitation showed that TLR4 was bound to RAGE and phospho-PKCβ_1_ (Fig. 5f), while phospho-PKCβ_1_ was bound to RAGE (Fig. 5g), indicating that RAGE, TLR4, and phospho-PKCβ_1_ were bound together structurally on the cell membrane.

In summary, the results of these cell experiments suggested that the RAGE-TLR4-PKCβ_1_ signal pathway might play an important role in the immune maturation of BMDCs induced by oxLDL plus AGEs. Also, RAGE, TLR4, and phospho-PKCβ_1_ were bound together structurally, on the cell membrane.

### LY333531 inhibited the activation of dendritic cells in diabetic ApoE^−/−^ mice and reversed the progression of atherosclerosis

LY333531 is a specific protein kinase C beta inhibitor, which can competitively and reversibly inhibit PKCβ1. The level of peripheral blood glucose was not significantly decreased in the LY333531 group, but the body weight of the mice was increased significantly (Additional file 1: Fig. S5a, b). LY333531 did not affect the blood lipid levels in the diabetic ApoE^−/−^ mice (Additional file 1: Fig. S5c–f), suggesting that LY333531 might have improved the condition of diabetic mice through ways other than glycemic control or decreasing blood lipid levels.

**Fig. 6:**
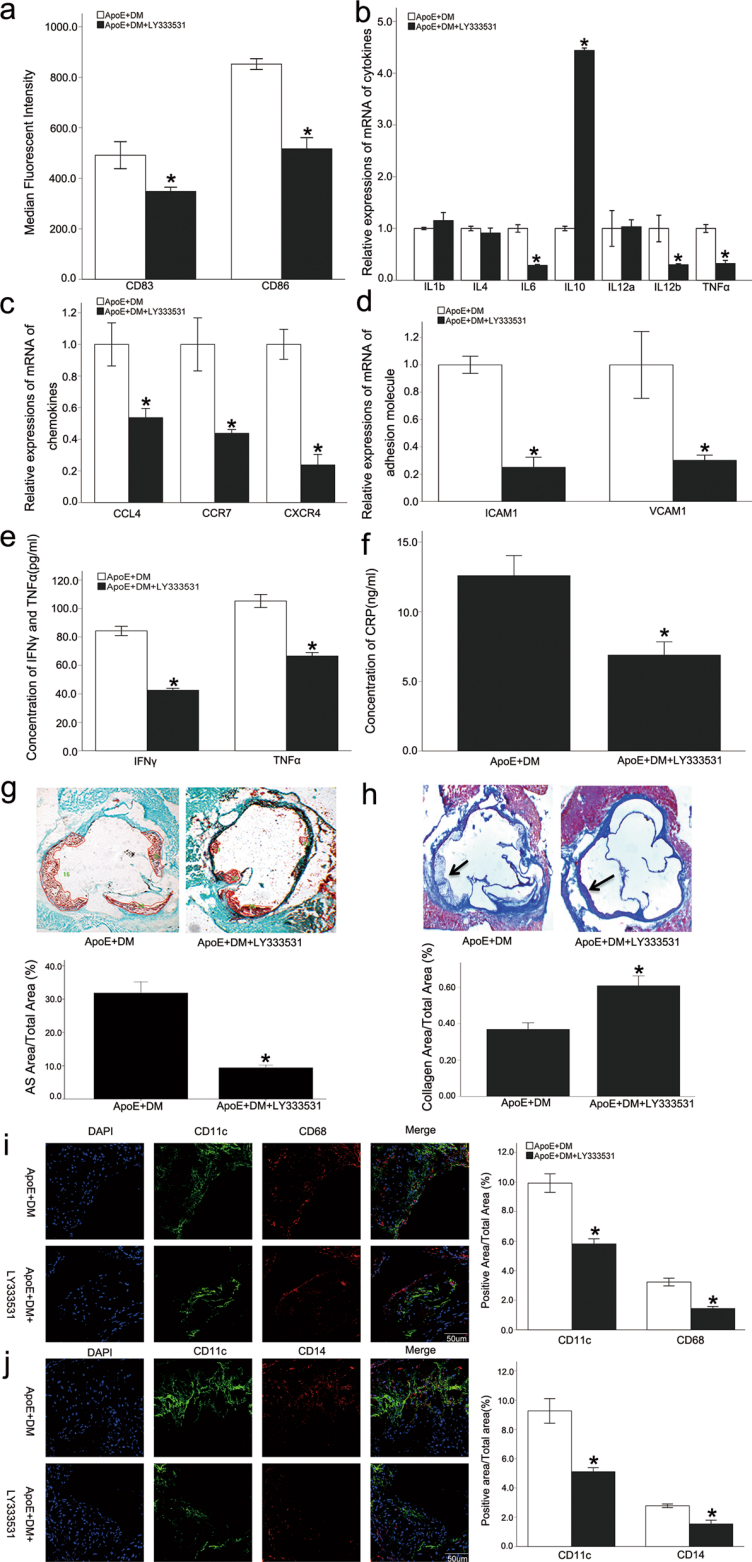
LY333531 inhibited the immune maturation of DCs and alleviated atherosclerosis in diabetic ApoE^−/−^ mice. The expression of the co-stimulatory molecules, CD83 and CD86, in splenic DCs was inhibited in the LY333531 group (**a**). The qPCR results also indicated that the expressions of TNFα, IL12b, IL6, CCL4, CCR7, and CXCR4 were inhibited in DCs, while the expression of IL10 was increased (**b**, **c**). In the LY333531 group, not only were the aortic adhesion molecules, ICAM1 and VCAM1, inhibited (**d**), but inflammatory markers such as TNFα, IFNγ, and CRP also were inhibited in peripheral blood (**e**, **f**). Systemic administration of LY333531 delayed the formation of aortic root plaques in mice with diabetic atherosclerosis when compared with the control group (**g**). Masson staining revealed that the collagen fiber composition in the plaques increased (**h**). Staining of cells in the plaque indicated that the distribution of inflammatory cells was decreased after LY333531 administration. Co-staining of CD68 and CD14 with CD11c showed that after excluding the influence of macrophages and monocytes, CD11c-labeled DCs had a significant reduction in plaques (**i**, **j**). Values, mean ± SED; n = 8; *p < 0.05 *vs.* ApoE + DM group; *DM* Diabetes mellitus, *IL* Interleukin, *ICAM* Intercellular adhesion molecule, *VCAM* Vascular cell adhesion molecule, *TNFα* Tumor necrosis factor alpha, *IFNγ* IFN gamma, *AS* Atherosclerosis, *DAPI* 4,6-diamino-2-phenyl indole

The expression of CD83 and CD86 in DCs isolated from spleens was inhibited significantly in the LY333531 group (Fig. 6a). The qPCR results also indicated that the expressions of TNFα, IL12b, IL6, CCL4, CCR7, and CXCR4 were significantly inhibited in DCs, while the expression of IL10 was increased (Fig. 6b, c). These results indicated that systemic PKCβ inhibition significantly suppressed the immune maturation of splenic DCs. In the LY333531 group, not only were the aortic adhesion molecules, ICAM1 and VCAM1, inhibited (Fig. 6d), but inflammatory markers such as TNFα, IFNγ, and CRP also were inhibited significantly in peripheral blood (Fig. 6e, f). These results indicated that the systemic administration of PKCβ inhibitor reduced the chronic low-grade systemic inflammation of diabetes mellitus.

Systemic administration of LY333531 significantly delayed the formation of aortic root plaques in mice with diabetic atherosclerosis when compared with the control group (Fig. 6g).

Masson staining revealed that the collagen fiber composition in the plaques increased (Fig. 6h). Staining of cells in the plaque indicated that the distribution of inflammatory cells was decreased after LY333531 administration. Since CD11c can be expressed on activated macrophages and monocytes, this study used CD68 and CD14 as markers for macrophages and monocytes, respectively, for co-staining with CD11c. The results showed that after excluding the influence of macrophages and monocytes, CD11c-labeled DCs had a significant reduction in plaques (Fig. 6i, j), suggesting that the overall stability of the plaque was enhanced.

## Discussion

Low-grade inflammation (Castelblanco et al. 2018; Zhang et al. 2019), which is critical in atherosclerosis development, has been reported to be an important feature in diabetes. DCs are antigen-presenting cells that play an important role in the inflammatory response. A previous study reported that a lack of DCs in mice could significantly reverse the progression of atherosclerotic plaques (Durpes et al. 2015). In further exploration of the subsets of DCs (Sun et al. 2020), it was found that CD11b^+^DC (Stoneman et al. 2007; Busch et al. 2014; Gao et al. 2016; Rombouts et al. 2016) and CCL17^+^DC (Rader and Daugherty, 2008; Weber et al. 2011) subsets have the effect of promoting atherosclerosis, and further exploration under more experimental conditions will provide multi-dimensional evidence. Whether CD103^+^DCs (Choi et al. 2011; Li et al. 2017; Clement et al. 2018) and plasmacytoid DCs (Daissormont et al. 2011; Macritchie et al. 2012) play pro-atherosclerotic or anti-atherosclerotic roles is controversial, which may depend on animal strains, different pathological models and other conditions. PKC is activated in diabetes (Liu et al. 2018) and is involved in a range of diabetic complications such as diabetic nephropathy and retinopathy (Chistiakov et al. 2019). Therefore, this study investigated whether the PKC signaling pathway was involved in the immune maturation of DCs in diabetic hyperlipidemia mice and the development of diabetic atherosclerosis.

**Fig. 7:**
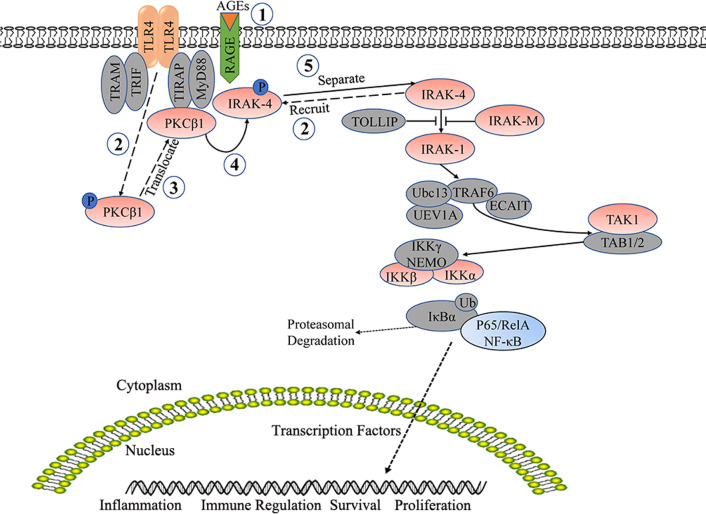
RAGE-TLR4-pPKCβ_1_ signal pathway diagram. After AGEs was combined with RAGE, the expression of RAGE was up-regulated and PKCβ_1_ was activated together with TLR4, phosphorylated PKCβ1 was transferred from the cytoplasm to the membrane, and formed RAGE-TLR4-pPKCβ_1_ complex, then activated the TLR4 signaling pathway through the phosphorylation of IRAK4, which promoted the phosphorylation of NF-κB, and further promoted the immune maturation of dendritic cells and the expression of inflammatory factors

The current study found that in diabetic ApoE^−/−^ mice, exposure to high glucose induced a DC-mediated chronic low-grade inflammatory response, which was related to the size and stability of the atherosclerotic plaques. Furthermore, AGEs induced the immune maturity of DCs in conjunction with stimulation by oxLDL. The possible mechanism involved the RAGE-TLR4-pPKCβ_1_ signaling pathway (Fig. 7). In addition, with PKCβ as the intervention target, LY333531 inhibited the DCs immune maturation that was induced by diabetic atherosclerosis, reduced the systemic chronic low-grade inflammatory response of diabetes mellitus, and stabilized and reduced the atherosclerotic plaques.

Paulson et al*.* found that DCs are activated and transformed in blood vessel intima into foam cells by phagocytosis of oxLDL, which promoted the development of atherosclerosis (Durpes et al. 2015). Meanwhile, in vivo studies (Blank et al. 2012) have found that both fluctuating and stable hyperglycemia can promote and enhance the differentiation and immune maturation of DCs. Furthermore, another in vitro experiment (Ge et al. 2005) confirmed that AGEs could induce the maturation of DCs and enhance immunity of DCs through increasing the expressions of scavenger receptor A and RAGE, which were mediated by Jnk pathway. Similarly, our study demonstrated that the expression of CD83 and CD86, which represent DC maturation, was up-regulated in the spleens of diabetic ApoE^−/−^ mice. Also, the expression of inflammatory cytokines (IL12b, IL4, and IL6), chemokine and chemokine receptors (CCL4, CCR7, and CXCR4) were significantly increased, indicating that diabetes plus hyperlipidemia could further induce immune maturation of DCs, and probably induced chronic low-grade inflammation that was mediated by DCs.

In the process of advanced atherosclerosis, DCs invade the atherosclerotic plaque from the adventitia along with vascular nourishment-related neovascularization, which leads to plaque instability and ulceration (Yilmaz et al. 2004). A previous study found that injection of diphtheria toxin into DTR^+/+^LDL^−/−^ mice induced apoptosis of DCs and reduced plaque formation by 55%, which enhanced plaque stability (Durpes et al. 2015). Therefore, it is hypothesized that the immune maturity of the DCs is related to the instability of the plaque. In the present study, the number of DCs and their immune maturation were significantly increased in diabetic ApoE^−/−^ mice, while the stability of the plaques decreased. When LY333531 was used to inhibit the DCs' immune maturation, the number of macrophages in the plaque significantly decreased, and the overall plaque stability was enhanced. These results extended the correlation between PKC and simple atherosclerosis to the occurrence and development of diabetic atherosclerosis.

The TLR-NF-κB signaling pathway is the primary inflammatory signaling pathway, and TLR activation also is required in the process of immune maturation of DCs (Alloatti et al. 2015). TLR4 is a TLR-associated protein that recognizes a range of endogenous ligands and activates the NF-κB signaling pathway, which promotes increased inflammatory cytokine gene expression in the atherosclerotic inflammatory process (Hayashi et al. 2012), while DC maturation via the NF-κB signaling pathway is well- known (Jung et al. 2020). In addition, TLR4 knockdown significantly down-regulates early atherosclerosis in diabetic ApoE^−/−^ mice (Lu et al. 2013), which may be related to monocyte activation (Bielinski et al. 2011) that is mediated by the TLR4 signaling pathway. In the present study, we found that the use of TLR4 inhibitors significantly down-regulated immune maturation of DCs and secretion of inflammatory cytokines induced by oxLDL plus AGEs. In addition, phosphorylation of IκB and NF-κB was significantly inhibited, which suggested that TLR4 was involved in the immune maturation of DCs.

Previous studies (Lin et al. 2011) indicated that selective inhibition of PKCα or PKCβ could inhibit the differentiation of CD14^+^ monocytes into macrophages or DCs in vitro and further inhibit their antigen-presenting function. Cejas et al*.* found that PKCβ was continuously activated during the differentiation of CD34^+^ bone marrow stem cells into DCs (Cejas et al. 2005). This effect could be enhanced by the PKC agonist, phorbol, and inhibited by PKC inhibitors (Davis et al. 1998; Cejas et al. 2005), suggesting that the PKC signaling pathway played an important role in the differentiation and immune maturation of DCs. However, the specific PKC subtype that is the main subtype affecting the differentiation and maturation of DCs is still controversial. Previous research reported that PKCα/δ/ε activation played a role in the activation of inflammatory cells through the TLR4 signaling pathway. The classical PKC signaling pathway in our study also was involved in the immune maturation process of DCs. Inhibition of PKCβ_1_ significantly down-regulated the expression of CD83, CD86, TNFα, and IFNγ induced by AGEs plus oxLDL or PMA. PKCα inhibitors and PKCβ_2_ inhibitors did not have the same effect, suggesting that the specific PKCβ_1_ subtype phosphorylation played an important role in the DCs immune maturation. Linghua et al*.* confirmed that the activation of PKCβ accelerated the process of diabetic atherosclerosis (Kong et al. 2013), and this study further confirmed the role of PKCβ1 and DC immune maturation in diabetic atherosclerosis.

AGEs have been shown to play a causative role in diabetic vascular disease, including atherosclerosis (Zhang et al. 2003). RAGE is a specific receptor for AGEs, which is expressed in low amounts in normal tissues but exhibits high levels of expression in AGEs-enriched regions of diabetic blood vessels (Yamagishi et al. 2003). RAGE plays a critical role in diabetic atherosclerosis through perpetuation of chronic vascular inflammation and through impairment of cholesterol metabolism (Bucciarelli et al. 2002; Wendt et al. 2006; Senatus et al. 2020). Blockade of RAGE stabilizes atherosclerotic lesion area in diabetic atherosclerosis mice (Bucciarelli et al. 2002), and deletion of *Ager* (the gene encoding RAGE) accelerates regression of diabetic atherosclerosis, at least in part through IRF7, which is verified in BMDMs (Senatus et al. 2020). RAGE recognizes AGEs and activates NF-κB (Ohtsu et al. 2017), but the intracellular domain of RAGE has only 43 amino acids, and there is no TIR-like homologous molecule for signal recognition. In this study, we confirmed that RAGE-TLR4-pPKCβ_1_ was a tripartite structure on the cell membrane. Ying Ju et al*.* demonstrated an NF-κB activation signaling pathway that was triggered by TLR4 and RAGE-regulated p38MAPK/JNK-activated PPARγ down-regulation in human osteoarthritis (OA) chondrocytes (Chen et al. 2013). This result explained the activation of the signal pathway of NF-κB in human OA chondrocytes by RAGE (Chen et al. 2013). Based on these results, this study proposed the hypothesis that when AGEs were bound to RAGE, this up-regulated the expression of RAGE and interacted with TLR4, which promoted PKCβ_1_ phosphorylation and formed a complex with RAGE-TLR4. After receptor ligation, TLR4 recruited MyD88 molecules. MyD88 further recruited IRAK4 and IRAK1 through its N-terminal death domain (DD). IRAK4 exerted kinase activity through autophosphorylation to phosphorylate IRAK1, which resulted in the activation of the latter and hyper-autophosphorylation. IRAK1 then dissociated from the complex and interacted with TRAF6 to finally activate the NF-κB signaling pathway. (Fig. 7) Thus, the expression of immune maturation and inflammatory factors in DCs were promoted, which might partially explain the mechanism by which hyperglycemia promotes the immune maturation of DCs. However, this possible mechanism needs further exploration.

There are several limitations associated with this study. First, specific inhibitors of PKCβ_1_ were not utilized in the in vivo experiments, which reduced the robustness of the experimental animal evidence. Second, repeated incubation failures occurred that resulted in our inability to include DTR^+^LDLR^−/−^ or DTR^−^LDLR^−/−^ transgenic mice in the current study. The unavailability of these transgenic mice led to a lack of direct in vivo evidence to support the relationship between DCs and atherosclerotic plaques. Third, our experimental design was based on type 1 diabetes, and it is necessary to conduct further research on type 2 diabetes. Fourth, the mechanism by which RAGE-TLR4-PKCβ_1_ bind together requires further exploration. Finally, this study only explored the typical PKC signaling pathways, but not other types of PKC signaling pathways.

## Conclusions

The present study demonstrated that diabetes mellitus aggravated chronic inflammation, and promoted atherosclerotic plaques in conjunction with hyperlipidemia, which at least in part through inducing the immune maturity of DCs. Compared with oxLDL, AGEs plus oxLDL further induced the immune maturation of DCs, and possibly through the RAGE-TLR4-pPKCβ_1_ signaling pathway. These results provided new insights for clinical prevention and treatment of diabetic atherosclerosis.

## Supplementary Information

**Additional file 1: Figure S1. **Fasting blood glucose and blood lipids level in mice were tested. **Figure S2.** Detection of CD11c^+^ cells purity by flow cytometry. **Figure S3.** The co-staining of PKC beta isoforms and RAGE with CD11c. **Figure S4.** Knockdown of PKCβ_1_ significantly inhibited the immune maturation of DCs. **Figure S5.** Plasma glucose, body weight and blood lipids were measured. **Table S1**. List of primers used in quantitative RT-PCR.

## Data Availability

The datasets during the current study are available from the corresponding author on reasonable request.

## Abbreviations

DM: Diabetes mellitus
AS: Atherosclerosis
DAPI: 4,6-Diamino-2-phenyl indole
IL: Interleukin
ICAM: Intercellular adhesion molecule
VCAM: Vascular cell adhesion molecule
TNFα: Tumor necrosis factor alpha
IFNγ: IFN gamma. CRP: C-reactive protein
oxLDL: Oxidized low density lipoprotein
AGEs: Advanced glycation end-products
NF-κB: Nuclear factor-κB
RAGE: Receptor for advanced glycation end products
pPKC: Phosphorylated protein kinase C
TLR4: Toll-like receptor 4
IRAK4: Interleukin receptor associated kinase 4
PKC: Protein kinase C
PMA: Phorbol ester
TG: Total triglyceride
TC: Total cholesterol
HDL-C: High-density lipoprotein cholesterol
LDL-C: Low-density lipoprotein cholesterol
DCs: Dendritic cells
BMDCs: Bone marrow-derived DCs
OCT: Optimal cutting temperature
GM-CSF: Granulocyte–macrophage colony-stimulating factor
OA: Osteoarthritis
DD: Death domain
