# Supplementary material for: Dendritic cell-mediated chronic low-grade inflammation is regulated by the RAGE-TLR4-PKCβ1 signaling pathway in diabetic atherosclerosis
Source: Mol Med. 2022 Jan 21;28:4. doi: 10.1186/s10020-022-00431-6 (PMC8780245; doi:10.1186/s10020-022-00431-6)
Supplement: Supplementary file 1 — Additional file 1: Figure S1. Fasting blood glucose and blood lipids level in mice were tested. Figure S2. Detection of CD11c+ cells purity by flow cytometry. Figure S3. The co-staining of PKC beta isoforms and RAGE with CD11c. Figure S4. Knockdown of PKCβ1 significantly inhibited the immune maturation of DCs. Figure S5. Plasma glucose, body weight and blood lipids were measured. Table S1. List of primers used in quantitative RT-PCR. [file 10020_2022_431_MOESM1_ESM.docx]

**Additional file 1: figure S1**

**Fasting blood glucose and blood lipids level in mice were tested.**


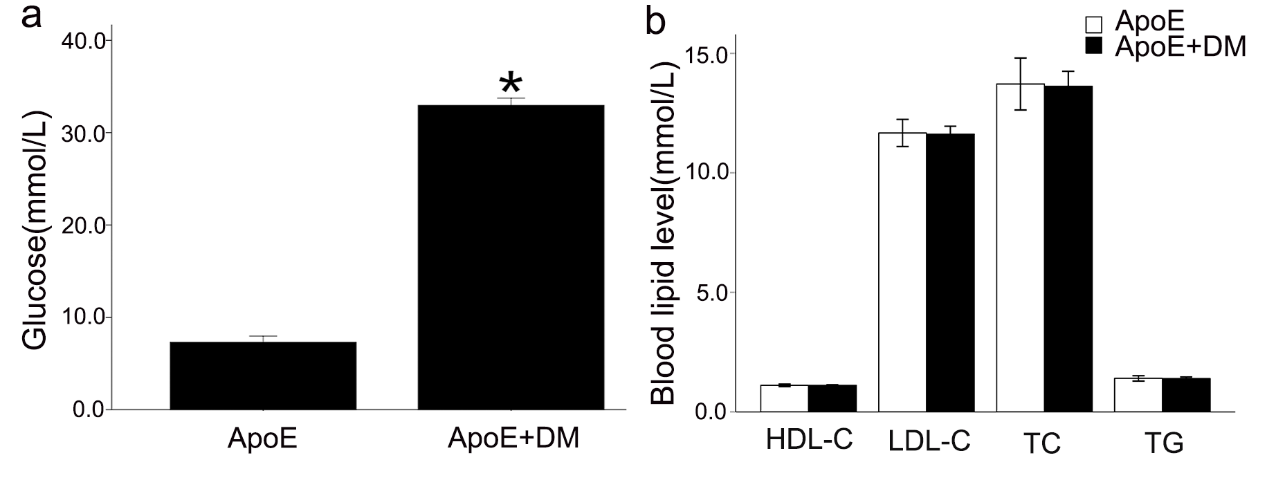


a: Plasma glucose measured; b: HDL-C, LDL-C, TC and TG monitored.

Values, mean±SED; n=8; * p < 0.05 vs. ApoE group; DM: Diabetes mellitus; TG: total triglyceride; TC: total cholesterol; HDL-C: high-density lipoprotein cholesterol; LDL-C: low-density lipoprotein cholesterol.

**Additional file 1: figure S2.**


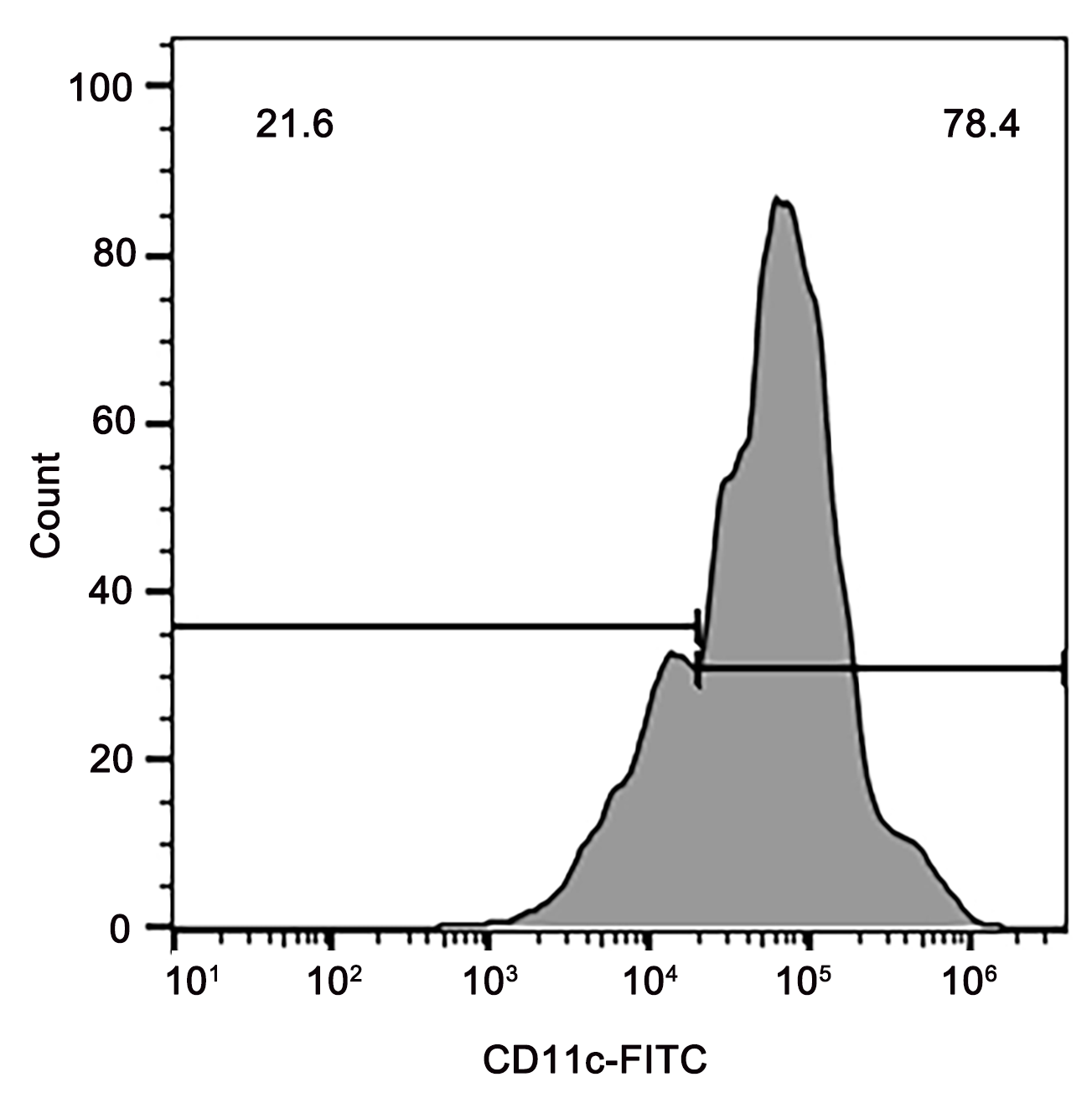


The number of CD11c^+^ cells detected by flow cytometry accounted for 78.4% of the total cells

**Additional file 1: figure S3.**


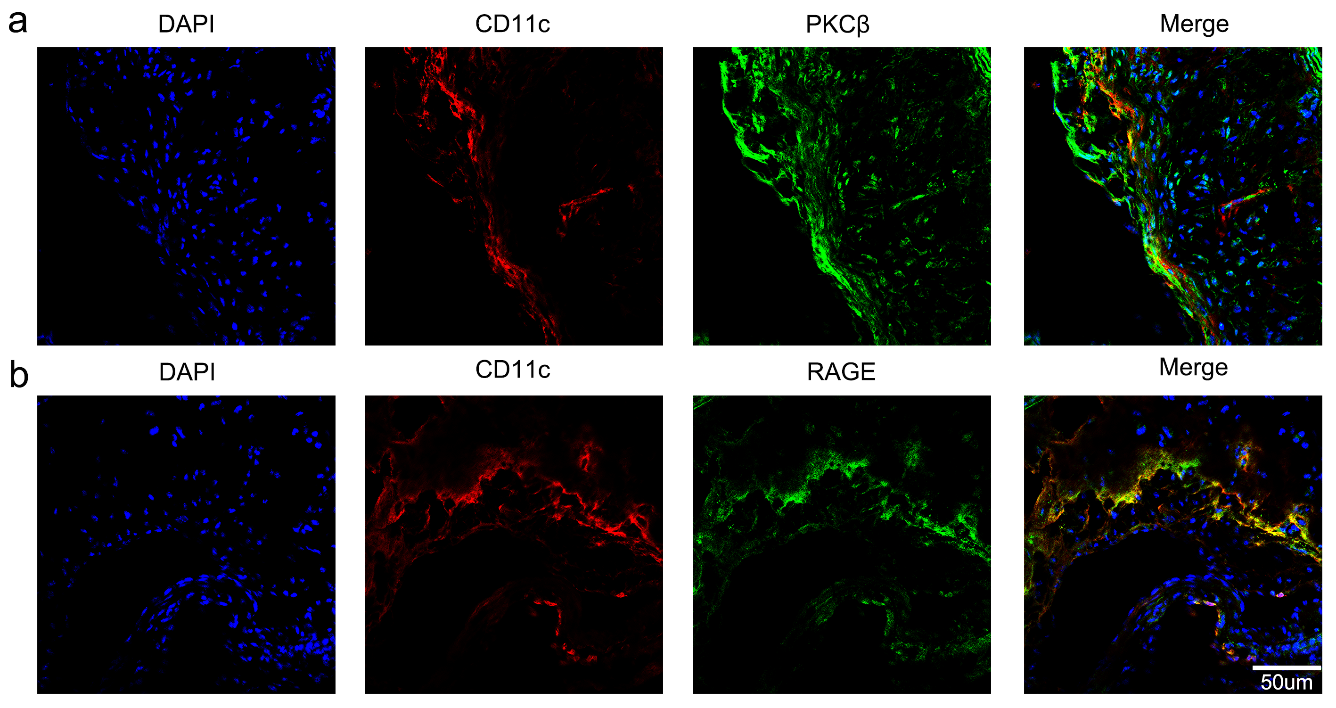


a-b: The co-staining of PKC beta isoforms and RAGE with CD11c.

DAPI:4,6-diamino-2-phenyl indole.

**Additional file 1: figure S4.**


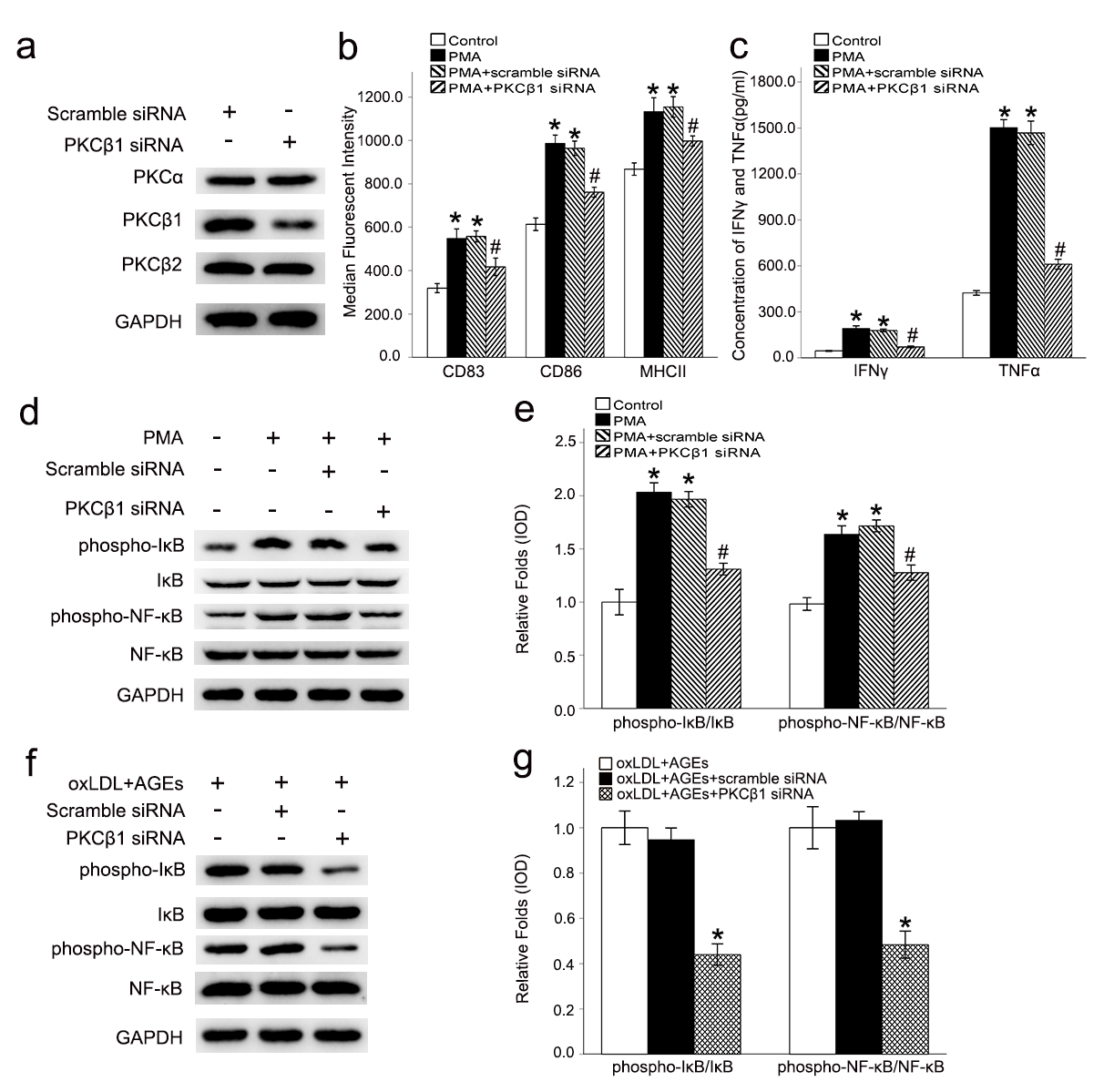


PMA, a PKC agonist, up-regulated the expression of CD83 and CD86 in BMDCs and promoted the secretion of the inflammatory cytokines, TNFα and IFNγ, while knockdown of PKCβ_1_ inhibited these effects (b and c, * p < 0.05 *vs.* control group; # p < 0.05 *vs.* PMA group). Knockdown of PKCβ_1_ inhibited the PMA- and oxLDL plus AGEs-induced activation of the NF-κB signaling pathway (d-g, * p < 0.05 *vs.* oxLDL+AGEs group).

Values, mean±SED; n=3, oxLDL: oxidized low density lipoprotein; AGEs: advanced glycation end-products; PKC: protein kinase C; IFNγ: IFN gamma; TNFα: Tumor necrosis factor alpha; PMA: phorbol ester; NF-κB: nuclear factor-κB.

**Additional file 1: figure S5.**

**
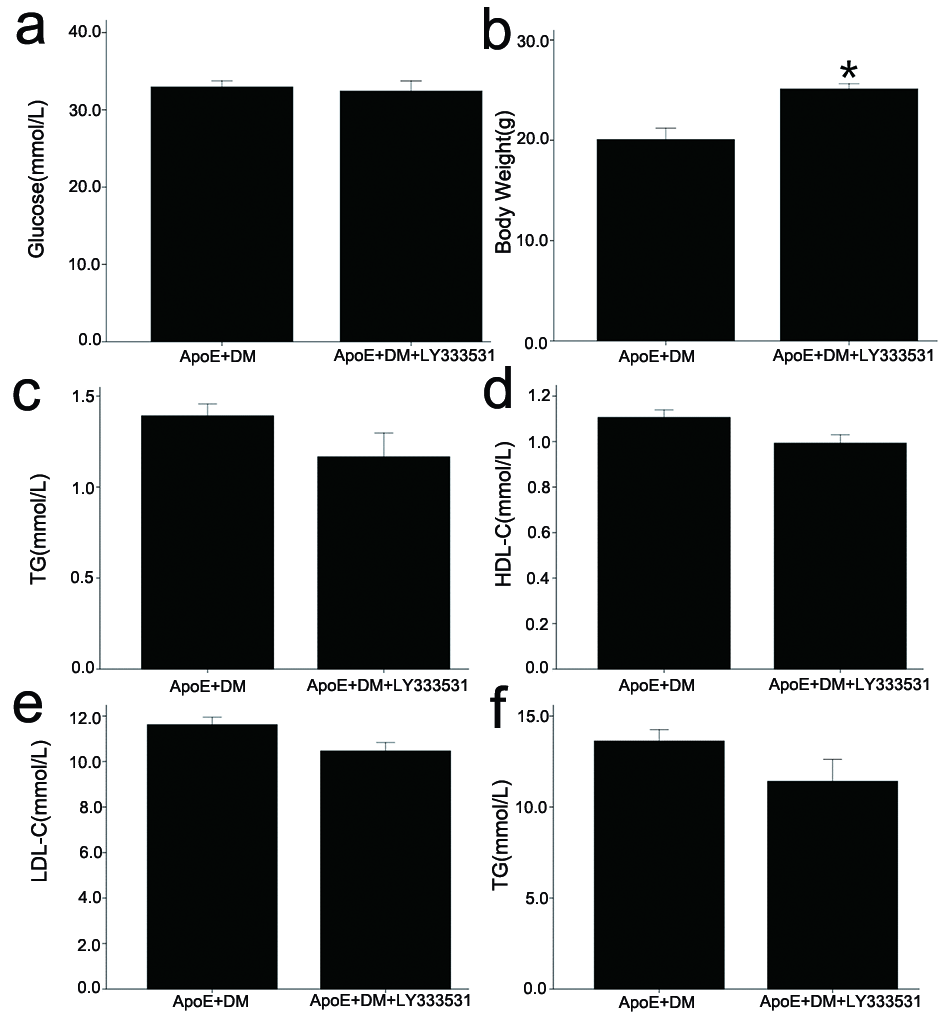
**

a-b: Plasma glucose monitored and Body weight measured; c-f: TG, HDL-C, LDL-C and TC monitored.

Values, mean±SED; n=8; * p < 0.05 vs. ApoE+DM group; DM: Diabetes mellitus; TG: total triglyceride; TC: total cholesterol; HDL-C: high-density lipoprotein cholesterol; LDL-C: low-density lipoprotein cholesterol.

**Additional file 1: table S1 : List of primers used in quantitative RT-PCR.**

| Genes | Primers（F-forward，R-Reverse） |
| --- | --- |
| IL10 | F-5’- GACCAGCTGGACAACATACTGCTAA-3’ |
|  | R-5’- GATAAGGCTTGGCAACCCAAGTAA-3’ |
| IL-12a | F-5’- TGTCTTAGCCAGTCCCGAAACC-3’ |
|  | R-5’- TCTTCATGATCGATGTCTTCAGCAG-3’ |
| IL-12b | F-5’- GCTCATGGCTGGTGCAAAGA-3’ |
|  | R-5’- GAGACGCCATTCCACATGTCA-3’ |
| IL-1b | F-5’- TCCAGGATGAGGACATGAGCAC-3’ |
|  | R-5’- GAACGTCACACACCAGCAGGTTA -3’ |
| IL-4 | F-5’- TCTCGAATGTACCAGGAGCCATATC -3’ |
|  | R-5’- AGCACCTTGGAAGCCCTACAGA-3’ |
| IL-6 | R-5’- CCACTTCACAAGTCGGAGGCTTA-3’ |
|  | F-5’- GCAAGTGCATCATCGTTGTTCATAC -3’ |
| TNFα | R-5’- GTTCTATGGCCCAGACCCTCAC-3’ |
|  | F-5’- GGCACCACTAGTTGGTTGTCTTTG-3’ |
| CCL4 | R-5’- CCATGAAGCTCTGCGTGTCTG-3’ |
|  | F-5’- GGCTTGGAGCAAAGACTGCTG-3’ |
| CCR7 | R-5’- GGTGGTGGCTCTCCTTGTCATT-3’ |
|  | F-5’-CTTCTTGAAGCACACCGACTCGTA -3’ |
| CXCR4 | R-5’- GTTGCCATGGAACCGATCA-3’ |
|  | F-5’- TGCCGACTATGCCAGTCAAGA-3’ |
| ICAM1 | R-5’- AACTGTGGCACCGTGCAGTC-3’ |
|  | F-5’- AGGGTGAGGTCCTTGCCTACTTG-3’ |
| VCAM1 | R-5’- TGCCGGCATATACGAGTGTGA-3’ |
|  | F-5’- CCCGATGGCAGGTATTACCAAG-3’ |
